# The neuroprotective action of dexmedetomidine on apoptosis, calcium entry and oxidative stress in cerebral ischemia-induced rats: Contribution of TRPM2 and TRPV1 channels

**DOI:** 10.1038/srep37196

**Published:** 2016-11-22

**Authors:** Hatice Akpınar, Mustafa Nazıroğlu, İshak Suat Övey, Bilal Çiğ, Orhan Akpınar

**Affiliations:** 1 Unit of Anesthesiology and Reanimation, Department of Oral and Maxillofacial Surgery, Faculty of Dentistry, University of Süleyman Demirel, Isparta, Turkey; 2 Department of Neuroscience, Institute of Health Science, University of Süleyman Demirel, Isparta, Turkey; 3 Center of Neuroscience, University of Süleyman Demirel, Isparta, Turkey; 4 Unit of Microbiology, Department of Oral and Maxillofacial Surgery, Faculty of Dentistry, University of Süleyman Demirel, Isparta, Turkey

## Abstract

Dexmedetomidine (DEX) may act as an antioxidant through regulation of TRPM2 and TRPV1 channel activations in the neurons by reducing cerebral ischemia-induced oxidative stress and apoptosis. The neuroprotective roles of DEX were tested on cerebral ischemia (ISC) in the cultures of rat primary hippocampal and DRG neurons. Fifty-six rats were divided into five groups. A placebo was given to control, sham control, and ISC groups, respectively. In the third group, ISC was induced. The DEX and ISC+DEX groups received intraperitoneal DEX (40 μg/kg) 3, 24, and 48 hours after ISC induction. DEX effectively reversed capsaicin and cumene hydroperoxide/ADP-ribose-induced TRPV1 and TRPM2 densities and cytosolic calcium ion accumulation in the neurons, respectively. In addition, DEX completely reduced ISC-induced oxidative toxicity and apoptosis through intracellular reactive oxygen species production and depolarization of mitochondrial membrane. The DEX and ISC+DEX treatments also decreased the expression levels of caspase 3, caspase 9, and poly (ADP-ribose) polymerase in the hippocampus and DRG. In conclusion, the current results are the first to demonstrate the molecular level effects of DEX on TRPM2 and TRPV1 activation. Therefore, DEX can have remarkable neuroprotective impairment effects in the hippocampus and DRG of ISC-induced rats.

Cerebral ischemia is a neurodegenerative disease that causes disability and mortality^1^,^2^. An accumulating body of evidence indicates that abnormalities of Ca^2+^ homeostasis is caused by excessive levels of free oxygen radicals in rats with cerebral ischemia^2^,^3^. In other words, there is mounting evidence to suggest that cell death after cerebral ischemia and spinal cord injury (SCI) is predominantly mediated by high amount of reactive oxygen species (ROS) and the subsequent over-production of poly (ADP-ribose [ADPR]) polymerase-1 (PARP-1), ultimately leading to mitochondrial dysfunction, release of apoptosis-inducing factors, and cell death^4^,^5^,^6^. Calcium ion (Ca^2+^) plays a crucial role in physiological activities, including apoptosis and mitochondrial functions^7^,^8^. Transient receptor potential (TRP) family is containing 8 subfamily. Melastatin and vanilloid subfamilies include TRP melastatin 2 (TRPM2) and TRP vanilloid 1 (TRPV1) Ca^2+^ permeable cation channels^4^,^9^. Capsaicin (CAP) is a pungent component of hot chili peppers, and TRPV1 is activated by different stimuli, including CAP 10 and oxidative stress^11^. A growing amount of evidence has shown that ADPR is produced as a byproduct of NAD metabolism. TRPM2 channel is activated by activation of ADPR pyrophosphatase enzyme in Nudix sequence of TRPM2 channel and the channel will be activated if the enzyme will be activated by different stimuli such as ADPR and oxidative stress^12^. The TRPM2 and TRPV1 channels are mainly expressed in the hippocampus and dorsal root ganglion (DRG)^13^,^14^,^15^. In addition, expression level of TRPM2 and TRPV1 in the brain were decreased by experimental brain injury^13^. As a result, ADPR production in the brains of cerebral ischemia-induced rats increased^16^. Prevention of apoptotic cascades through inhibition of the TRPM2 and TRPV1 in the hippocampal (HIPPO) and DRG neurons of rats were more recently reported^5^,^17^. These later results raised the possibility that HIPPO and DRG neuron apoptosis mediated by the TRPM2 and TRPV1 channels could also contribute to cerebral ischemia injuries, as accumulating evidence suggests their involvement in cerebral ischemia and SCI, including oxidative stress induced by the TRPM2 and TRPV1 channels^2^,^5^.

Dexmedetomidine (DEX) is an important drug for long-term sedation in intensive care patients because it induces a rapid response and is easily controllable. In addition to its sedative effect, DEX plays an important role in the treatment of pain and SCI 1,18. Evidence has emerged indicating that the effect DEX has on ion channels may be an important mechanism underlying DEX-induced peripheral anti-nociception^1^. The results of recent studies show that DEX decreased cerebral ischemia and SCI-induced intracellular ROS production and apoptosis in the brain and DRG of rat, respectively^1^,^19^. Recently, it has been demonstrated that DEX potently inhibits overload Ca^2+^ entry through voltage gated calcium channels (VGCC) and glutamate (NMDA) receptors in the DRGs and hippocampus of rats^20^,^21^,^22^. Therefore, DEX may reduce the entry of overload Ca^2+^ via modulation of TRPM2 and TRPV1 channel activations in the HIPPO and DRG neurons of rats with cerebral ischemia, and this effect should be clarified.

Accumulating reports in the drug discovery potential of TRP channels provides continual interest for the hypothesis that block of TRP channel inhibition underlies many of the treatments related with SCI and cerebral ischemia-induced brain injuries^5^,^23^. However, there have been no reports on overload Ca^2+^ entry via activations of TRPM2 and TRPV1 in rats with cerebral ischemia. To address this interaction between DEX, TRPM2 and TRPV1, we investigated the protective actions of DEX treatment on apoptosis, oxidative stress levels, Ca^2+^ entry values as well as involvement of TRPM2 and TRPV1 activations on the values in the DRG and HIPPO neurons in rat with cerebral ischemia.

## Results

### The effects of DEX treatment on [Ca^2+^]_i_ accumulation through TRPM2 activation in the HIPPO and DRG neurons of rats with cerebral ISC

**Figure 1:**
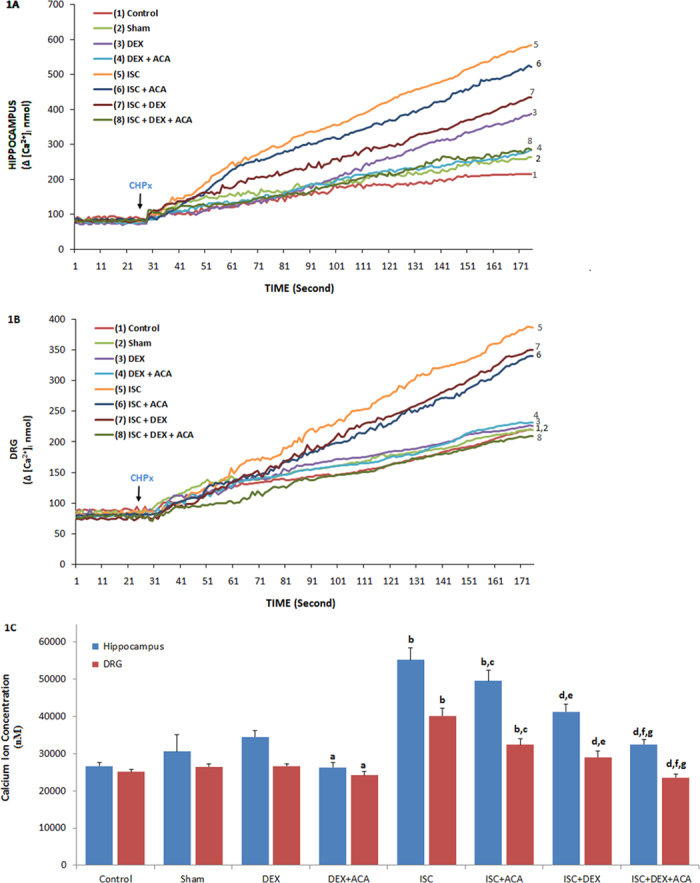
Effects of dexmedetomidine (DEX) treatment on [Ca^2+^]_i_ through TRPM2 channel in HIPPO (Fig. 1A) and DRG (Fig. 1B) neurons in control and rats with cerebral ischemia (ISC) (n = 12 and mean ± SD). The animals received intraperitoneal DEX (40 mg/ml at 3th, 24^th^ and 48^th^ hours of cerebral ischemia. These neurons were dissected from control and treated animals for further treating TRPM2 agonist, cumene hydroperoxide (CHPx and 1 mM) in the presence of normal extracellular calcium (1.2 mM) for 175 seconds. The TRPM2 channels in the neurons were inhibited by ACA (0.025 mM). (^a^p ≤ 0.05 versus (vs) DEX group. ^b^p ≤ 0.001 vs control, sham and DEX groups. ^c^p ≤ 0.05 and ^d^p ≤ 0.001 vs ISC group. ^e^p ≤ 0.05 and ^f^p ≤ 0.001 vs ISC+ACA group. ^g^p ≤ 0.05 vs ISC+DEX group) (Fig. 1C).

The best TRPM2 channel antagonist within nonspecific agonists of the TRPM2 channel is N-(p-amylcinnamoyl) anthranilic acid (ACA)^24^ and we used with the potential treatment action of DEX in the ISC-induced HIPPO and DRG neuron injury models through the TRPM2 involved in Ca^2+^ accumulation. For the aim, these neurons were dissected from control and treated animals and they were further *in vitro* treated with TRPM2 agonist CHPx although they were inhibited by ACA. The [Ca^2+^]_i_ concentration in the HIPPO (Fig. 1A) and DRG (Fig. 1B) neurons was markedly (p ≤ 0.05) higher in the DEX group than in the DEX+ACA group. As compared to the control, sham, and DEX groups, the [Ca^2+^]_i_ was importantly (p ≤ 0.001) high in the ISC group. The [Ca^2+^]_i_ was also significantly (p ≤ 0.05) lower in the ISC+ACA group than in the ISC group. Compared to the ISC (p ≤ 0.001) and ISC+ACA (p ≤ 0.05) groups, the [Ca^2+^]_i_ concentration was also further (p ≤ 0.05) decreased in the ISC+DEX and ISC+DEX+ACA groups by DEX and ACA treatments.

### The involvement of DEX treatment on [Ca^2+^]_i_ concentration, TRPM2 and TRPV1 activations in the DRGs of rats with cerebral ischemia

**Figure 2:**
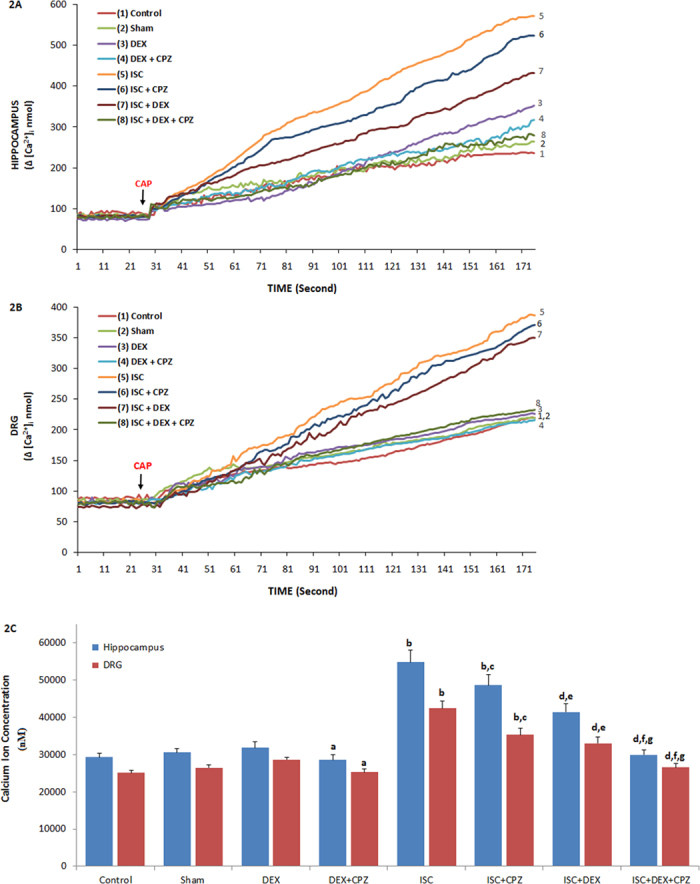
Effects of dexmedetomidine (DEX) treatment on [Ca^2+^]_i_ through TRPV1 channel in HIPPO (Fig. 2A) and DRG (Fig. 2B) neurons in control and rats with cerebral ischemia (ISC) (n = 12 and mean ± SD). The animals received intraperitoneal DEX (40 mg/ml at 3^th^, 24^th^ and 48^th^ hours of cerebral ischemia. These neurons were dissected from control and treated animals. Fura-2-loaded rat HIPPO neurons were further treated with TRPM2 agonist capsaicin (CAP and 0.01 mM) in the presence of normal extracellular calcium (1.2 mM) for 175 seconds. The TRPM2 channels in the neurons were inhibited by capsazepine (CPZ and 0.1 mM). (^a^p ≤ 0.05 versus (vs) DEX group. ^b^p ≤ 0.001 vs control, sham and DEX groups. ^c^p ≤ 0.05 and ^d^p ≤ 0.001 vs ISC group. ^e^p ≤ 0.05 and ^f^p ≤ 0.001 vs ISC+CPZ group. ^g^p ≤ 0.05 vs ISC+DEX group) (Fig. 1C).

Although CAP is a TRPV1 receptor agonist^10^,^15^, capsazepine (CPZ) is a specific TRPV1 receptor antagonist. The animals received intraperitoneal DEX (40 mg/ml) at 3th, 24^th^ and 48^th^ hours of cerebral ischemia. In order to identify the TRPV1 receptor involved in Ca^2+^ accumulation in the HIPPO and DRG neurons, these neurons were dissected from control and treated animals and they were further *in vitro* treated with TRPV1 agonist CAP but they were further inhibited by CPZ (Fig. 2A,B,C). The [Ca^2+^]_i_ concentrations in the HIPPO and DRG neurons were high (p ≤ 0.05) in the DEX group as compared to the DEX+CPZ group. The [Ca^2+^]_i_ was markedly (p ≤ 0.001) higher in the ISC group than in the control, sham, and DEX groups. As compared to the ISC group, the [Ca^2+^]_i_ concentration was also low (p ≤ 0.05) in the ISC+CPZ group. Compared to the ISC (p ≤ 0.001) and ISC+CPZ (p ≤ 0.05) groups, the [Ca^2+^]_i_ was also further (p ≤ 0.05) decreased by CPZ treatments in the ISC+DEX and ISC+DEX+ACA groups.

### The effects of DEX on ADPR-induced TRPM2 current densities in the DRGs of cerebral ISC-induced rats

**Figure 3:**
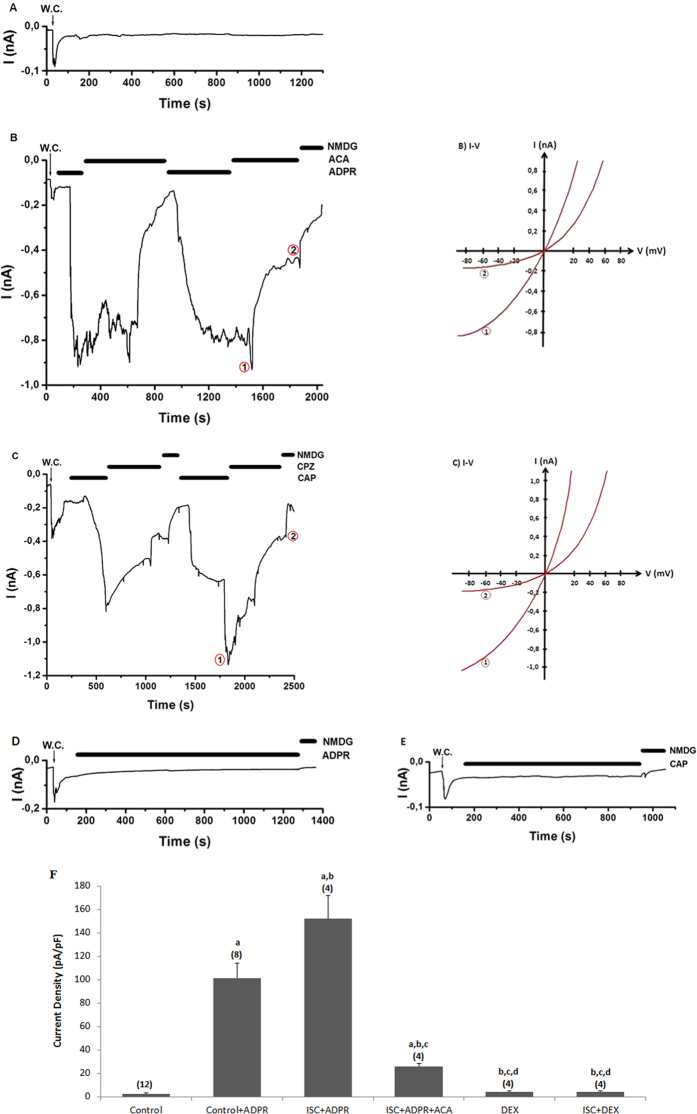
Effects of dexmedetomidine (DEX) treatment on TRPM2 channel activation in the DRG of in control and cerebral ischemia (ISC)-induced rats. The holding potential was minus sixty millivolt in the analyses. (**A**) Control: Original recordings from control neuron. (**B**) Control+ADPR group: DRG isolated from rats of control and sham groups without ISC induction and they were stimulated by capsaicin (0.01 mM) and ADPR (1 mM) but inhibited by ACA (0.025 mM). (**C**) ISC+ADPR group: TRPM2 currents in the DRG neurons of ISC-induced rats were gated by ADPR (1 mM) in the patch pipette and they were inhibited by ACA in the bath of patch chamber. (**D**) DEX+ADPR group: The rats received intraperitoneal DEX and then the DRG neurons were stimulated by *in vitro* ADPR (1 mM in patch pipette). (**E**) ISC+DEX+ADPR group: The rats received DEX at 3^rd^, 24^th^ and 48^th^ hours after induction of experimental ISC and then the DRG was stimulated by ADPR (1 mM). (**B**) I-V and (**C**) I-V: Current voltage relationships and they are same experiments as in panels B and C, respectively. W.C. is whole-cell. (**F**) TRPM2 channel capacitance of the DRG in control and ISC-induced rats (mean ± SD). The DRG neurons were further treated *in vitro* afterwards with ADPR (1 mM) and ACA (0.025 mM). Capacitance calculation of the currents was described in the method section. The numbers of group were indicated in parentheses. (^a^p ≤ 0.001 versus control. ^b^p ≤ 0.001 versus control+ADPR group. ^c^p ≤ 0.001 versus control+ADPR+ACA group. ^c^p ≤ 0.001 versus ISC+ADPR group. ^e^p ≤ 0.001 versus ISC+ADPR+ACA group).

The TRPM2 currents induced by ADPR delayed gradually (within 3.43 ± 1.95 minutes) following the infusion of ADPR into the cytosol of the neurons (Fig. 3B). The delay between the current onset and current amplitude of 0.84 nA during the stimulation with ADPR.

The ADPR currents were reversibly blocked by TRPM2 blocker (ACA) and a substitute ion (NMDG^+^) for Na^+^ (see Fig. 3B,C). Control records were taken every day from same animals that were used to study TRPM2. We observed no currents in control records when it was not activated by intracellular ADPR (1 mM) of path-pipette (Fig. 3A). Compared to control mean the current densities (as pA/pF) values in the DRG, the current densities increased in groups of control+ADPR and ISC+ADPR. The ACA treatments markedly (p ≤ 0.001) decreased the mean values in the control+ADPR+ACA and control +ADPR+ACA groups (Fig. 3F). In addition, TRPM2 channels were fully blocked by DEX treatment, and the currents were (p ≤ 0.001) lower in the DEX and ISC+DEX groups as compared to the control+ADPR and ISC+ADPR groups.

### The protective actions of DEX on TRPV1 current densities in the DRGs of rats with cerebral ischemia

**Figure 4:**
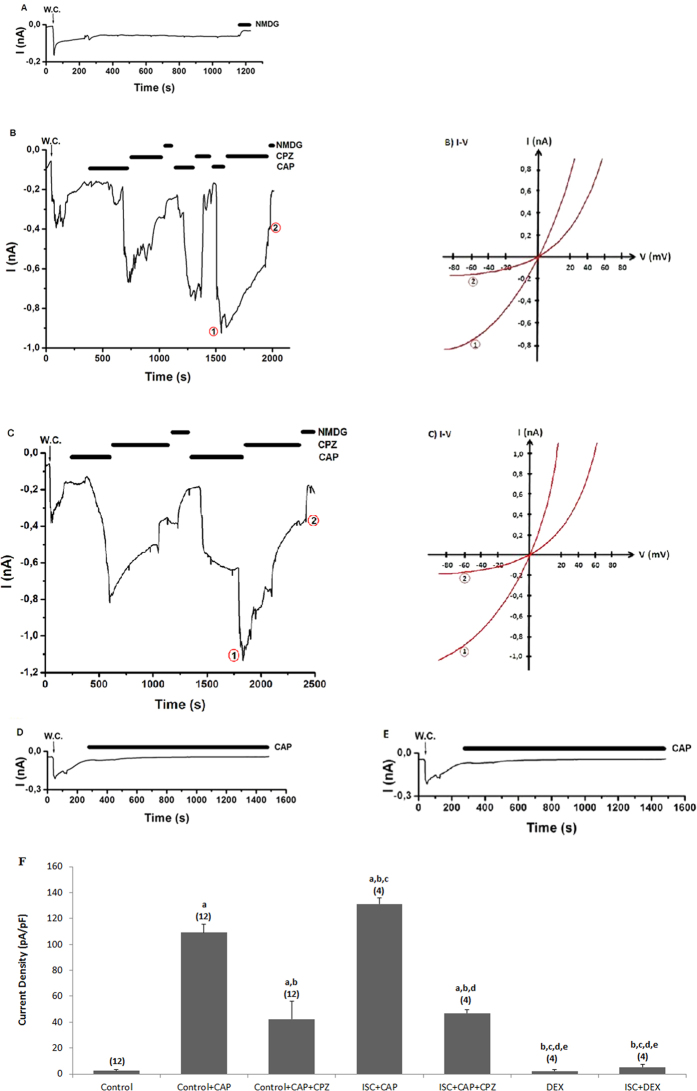
Effects of dexmedetomidine (DEX) treatment on TRPV1 channel in the DRG of in control and cerebral ischemia (ISC)-induced rats. The holding potential was minus sixty millivolt in the analyses. (**A**) Control: Original recordings from control neuron. (**B**) Control+CAP group: DRG isolated from rats of control and sham groups without ISC induction and they were stimulated by capsaicin (CAP and 0.01 mM) but inhibited by CPZ (0.1 mM) in the bath of patch chamber. (**C**) ISC+CAP group: TRPV1 currents in the DRG neurons of ISC-induced rats were gated by CAP (0.01 mM) and they were inhibited by CPZ (0.1 mM). (**D**) DEX+CAP group: The rats received intraperitoneal DEX and then the DRG neurons were stimulated by *in vitro* CAP (0.01 mM). (**E**) ISC+DEX+CAP group: The rats received DEX at 3^rd^, 24^th^ and 48^th^ hours after induction of experimental ISC and then the DRG was stimulated by ADPR (1 mM). (**B**) I-V and (**C**) I-V: Current voltage relationships and they are same experiments as in panels B and C, respectively. (**F**) TRPV1 channel capacitance of the DRG in control and ISC-induced rats. For each of the applications, the initial current density was calculated after administration of CAP as described in the method section (mean ± SD). The numbers of group were indicated in parentheses. (^a^p ≤ 0.001 vs control. ^b^p ≤ 0.001 vs control+CAP group. ^c^p ≤ 0.001 vs control+CAP+CPZ group. ^d^p ≤ 0.001 vs ISC+CAP group. ^e^p ≤ 0.001 vs ISC+CAP+CPZ group).

Control record is shown in Fig. 4A and it was taken from the DRG neurons when it was not stimulated by CAP. The current densities of DRG about 0.88 nA were induced by addition of TRPV1 channel agonist (CAP and 0.01 mM) into patch-chamber within 1.33 ± 0.28 minute (Fig. 4B). However, these currents were approximately returned to control levels by addition of CPZ into patch-chamber (see Fig. 4B,C). On the other word, these currents were markedly (p ≤ 0.001) decreased in the control+CAP and ISC+CAP groups by CPZ treatments. Therefore, we observed a TRPV1 activating role for cerebral ischemia induction in the neurons. However, DEX induced protective actions on the current densities and the TRPV1 currents were also significantly (p ≤ 0.001) lower in the DEX+CAP and ISC+DEX+CAP groups than in the ISC-only group (Fig. 4F), and the values were decreased to control levels by DEX treatments.

### Protective role of DEX on values of apoptosis, MTT, caspase in the HIPPO neuron of rats with cerebral ischemia

**Figure 5:**
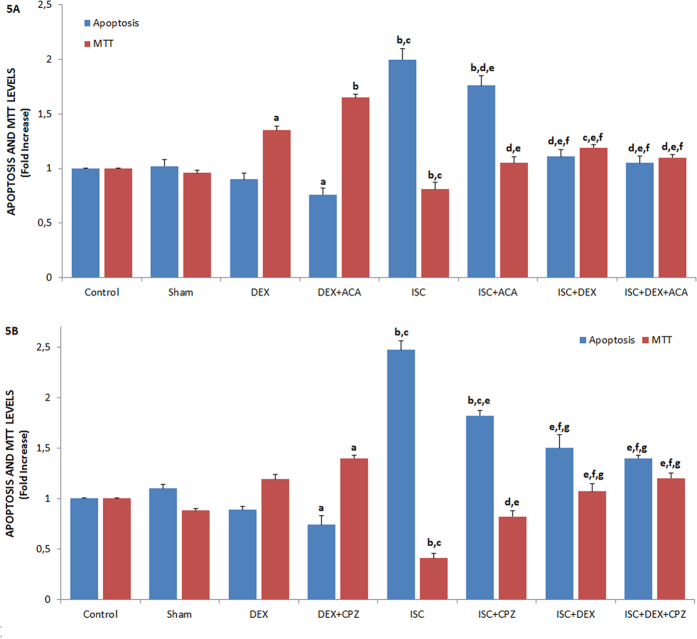
Effects of dexmedetomidine (DEX) treatments on apoptosis and MTT levels through TRPV1 and TRPM2 channel activities in HIPPO neurons of ischemia (ISC)-induced rats [mean ± SD and n = 12 except control (n = 8)]. The HIPPO neurons were further treated *in vitro* afterwards with CHPx (1 mM) (Fig. 5A) and CAP (0.01 mM) (Fig. 5B) although they were inhibited by ACA (0.025 mM) and CPZ (0.1 mM) and, respectively. Values were expressed as fold-increase (experimental/control). (^a^p ≤ 0.05 and ^b^p ≤ 0.001 vs control, sham and DEX groups. ^c^p ≤ 0.001 and ^d^p ≤ 0.05 vs DEX+ACA and DEX+CPZ group. ^e^p ≤ 0.001 vs ISC group. ^f^p ≤ 0.05 vs ISC+ACA and ISC+CPZ group. ^g^p ≤ 0.05 vs ISC+DEX group).

**Figure 6:**
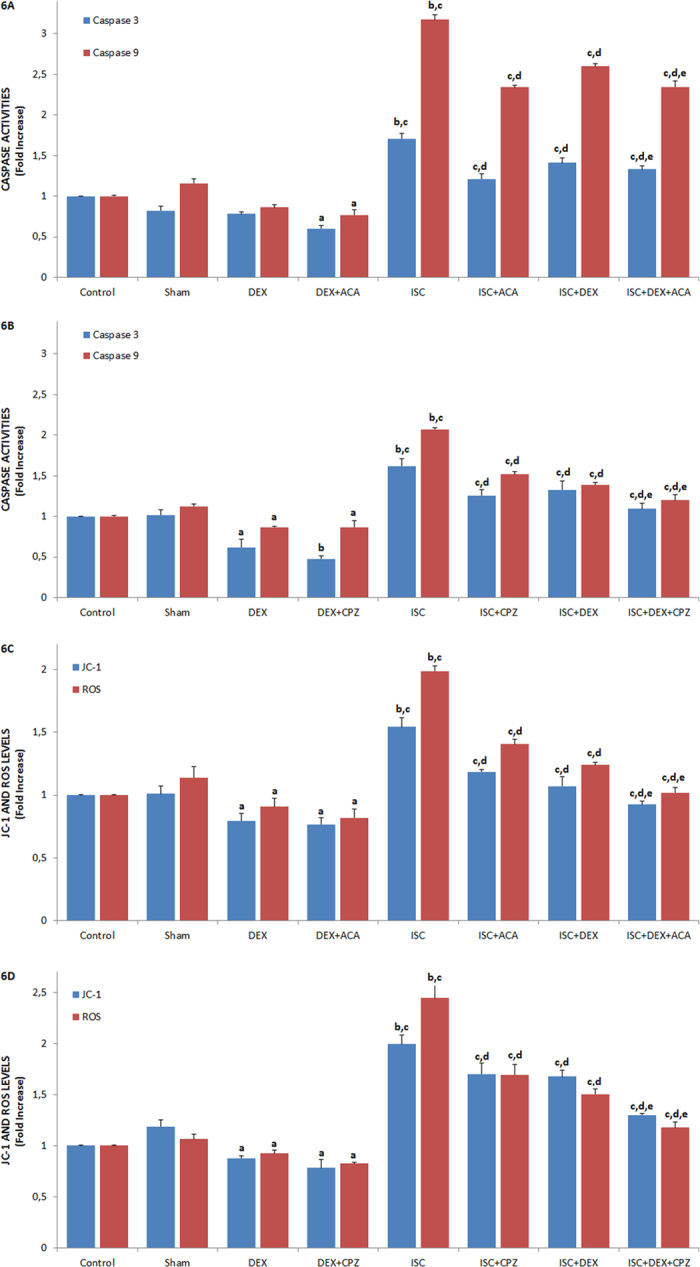
Effects of dexmedetomidine (DEX) treatments on caspase 3, caspase 9 activities, mitochondrial membrane depolarization (JC-1) and intracellular ROS production through TRPV1 and TRPM2 channel activities in HIPPO neurons of ischemia (ISC)-induced rats [mean ± SD and n = 12 except control (n = 8)]. HIPPO neurons of control, sham, DEX, DEX+ACA, ISC, ISC+ACA, ISC+DEX and ISC+DEX+ACA groups were *in vitro* stimulated in TRPM2 (6A and C) and TRPV1 (6B and D) experiments by CHPx (1 mM) and CAP (0.01 mM) although they were inhibited by ACA (0.025 mM) and CPZ (0.1 mM) respectively. (^a^p ≤ 0.05 and ^b^p ≤ 0.001 vs control, sham and DEX groups. ^c^p ≤ 0.001 vs DEX+ACA and DEX+CPZ groups. ^d^p ≤ 0.001 vs ISC group. ^e^p ≤ 0.05 vs ISC+DEX group).

Figures 5 and 6 show the effects of DEX on cerebral ischemia-induced apoptosis, MTT levels, and caspase 3 and 9 activities through TRPM2 (Figs 5A and 6A) and TRPV1 (Figs 5B and 6B) channel activation in the HIPPO neurons. As compared to the control, sham, and DEX groups, the level of apoptosis, activities of caspase 3 and 9 were high (p ≤ 0.001) in the ISC groups although the MTT levels were low (p ≤ 0.001). The cerebral ischemia-induced apoptosis levels, caspase 3, and caspase 9 activities significantly decreased in the ISC+ACA (p ≤ 0.05), ISC+CPZ (p ≤ 0.05), ISC+DEX (p ≤ 0.001), ISC+DEX+ACA (p ≤ 0.001), and ISC+DEX+CPZ (p ≤ 0.001) groups through the ACA, CPZ, and DEX treatments, although MTT levels in the groups were markedly (p ≤ 0.05 and p ≤ 0.001) increased by the treatments.

### Protective role of DEX on mitochondrial membrane depolarization (JC-1) and intracellular ROS production levels in the HIPPO and DRG neurons of rats with cerebral ischemia

The protective actions of DEX on JC-1 and ROS levels in the HIPPO neurons through TRPM2 were shown in Fig. 6C,D, respectively. As compared to control and sham control groups, the JC-1 and ROS levels in the HIPPO and DRG (data are not shown) neurons were high (p ≤ 0.001) in the ISC groups. However, the JC-1 and ROS level of rat HIPPO neurons were low in the ISC+ACA (p ≤ 0.05), ISC+CPZ (p ≤ 0.05), ISC+DEX (p ≤ 0.001), ISC+DEX+ACA (p ≤ 0.001), and ISC+DEX+CPZ (p ≤ 0.001) groups as compared to ACA, CPZ, and DEX treated groups, whereas MTT levels in the groups were high (p ≤ 0.05 and p ≤ 0.001) in the treated groups.

### Results of PARP and procaspase 3 and 9 expressions in HIPPO neurons

**Figure 7:**
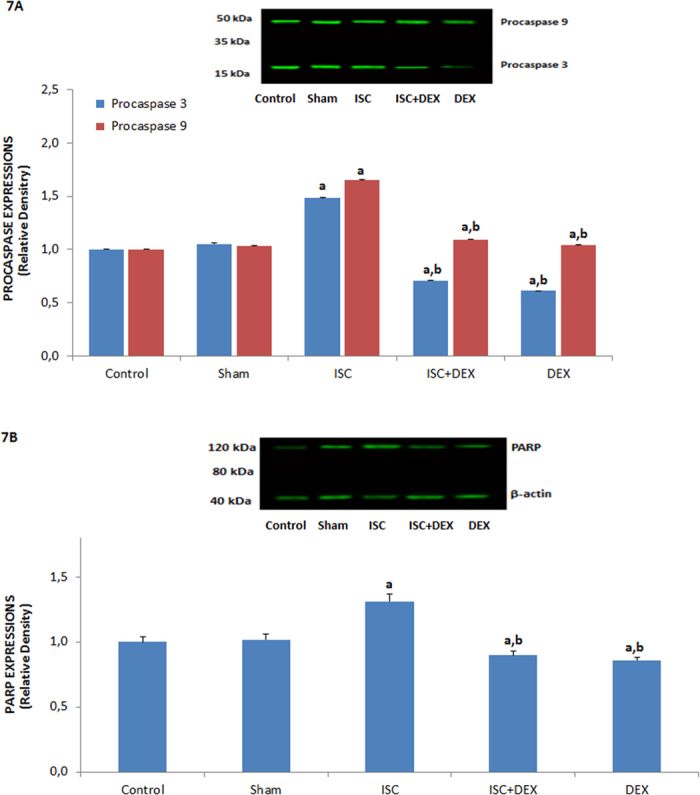
Protective roles of dexmedetomidine (DEX) treatment on procaspase 3, procaspase 9 (7 A) and PARP (7B) activities in HIPPO neuron of ischemia (ISC)-induced rat. Values are presented as mean ± SD of 3 separate experiments and expressed relative density over the control. (^a^p ≤ 0.05 vs control and sham groups. ^b^p ≤ 0.001 vs ISC group).

Procaspase 3 and 9 are main sources of active caspase 3 and 9 in the executioner caspase-activated pathways and mitochondrial apoptotic cascades, respectively^25^,^26^,^27^. As indicators of apoptosis, procaspases 3 and 9 were assayed in the current study (Fig. 7A). The expressions of procaspases 3 and 9 in the HIPPO and DRG (data are not shown) neurons were high (p ≤ 0.05) in the ISC group as compared to the control and sham groups, these expressions were modulated in the DEX and ISC+DEX groups by the DEX treatments (p ≤ 0.05).

The enzyme PARP is abundant in neurons that are indicating and signaling DNA damage to repair mechanisms. After being activated in response to single-strand DNA breaks PARP subsequently attaches to regions of injured DNA^4^,^26^. The activation of TRPM2 is caused by PARP-induced ADPR and NAD^+^ pathways^12^, and we observed TRPM2 channel activation in the current study. In addition, PARP expressions in the HIPPO neurons were markedly (p ≤ 0.05) high in the ISC group as compared to the control and sham groups (Fig. 7B). However, PARP activities in the neurons were significantly lower in the DEX and ISC+DEX groups than in the ISC (p ≤ 0.001) and control (p ≤ 0.05) groups.

## Discussion

**Figure 8:**
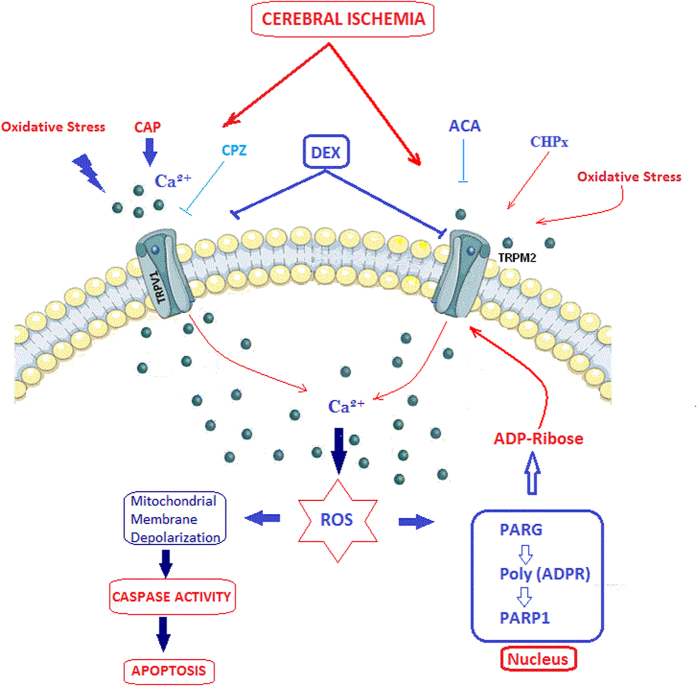
Possible molecular pathways of involvement of DEX in cerebral ischemia-induced apoptosis, oxidative stress and calcium accumulation through TRPM2 and TRPV1 channels in the HIPPO and DRG neurons of rats. The TRPM2 channel is activated by ADP-ribose (ADPR) and oxidative stress although it is inhibited by ACA. The TRPV1 channel is activated by oxidative stress and capsaicin (CAP) and it is blocked by capsazepine (CPZ). Increased intracellular Ca^2+^ concentration through TRPM2 and TRPV1 channels in cerebral ischemia (ISC) may lead to neuronal toxicity, reactive oxygen species (ROS), inflammatory processes and eventual cell death. DEX reduced oxidative stress, apoptotic factors (including caspase 3 and 9), mitochondrial membrane depolarization and Ca^2+^ influx through the inhibition of TRPM2 and TRPV1 channel activation.

For the long- and short-term sedations, DEX is used as an analgesic and anesthetic. In addition to its effectiveness in sedation, DEX reportedly has a regulatory effect on VGCC and oxidative stress in neurons^20^,^21^,^22^. Excessive production of mitochondrial ROS and an overload of Ca^2+^ through increased TRPM2 and TRPV1 channel activity are two of the main causes of neurodegenerative diseases in hippocampus and induction of peripheral pain in DRG (Fig. 8)^5^,^10^,^17^. The aim of the study was to evaluate whether DEX functionally interacts with TRPM2 and TRPV1 in the HIPPO and DRG neurons of rats with cerebral ischemia. We observed that activations of TRPM2 and TRPV1 channels are potentially inhibited in the neurons by the DEX treatment. Therefore, we suggest that DEX involved changes of Ca^2+^ entry through activations of TRPM2 and TRPV1 channels and the activations resulted in production of mitochondrial membrane depolarization-induced free oxygen radical in cerebral ischemia-induced brain HIPPO neuronal injury and pain induction.

Free oxygen radical acts a main action in the development of cerebral ischemia^1^,^28^. Accumulating evidences indicated that activations of TRPM2 and TRPV1 channels are increased in brain injury-induced mitochondrial activation and excessive Free oxygen radical production by the excessive mitochondrial Ca^2+^ uptake^6^,^9^,^29^. Furthermore, DEX induces antioxidant effects in the spinal cord (DRG) injuries and cerebral ischemia (hippocampus) of rats^1^,^18^,^19^. Intracellular ROS production was used as an indicator of oxidative stress in the HIPPO neurons. As a result, the cerebral ischemia-induced ROS production was strongly decreased by DEX treatment because of inhibition of overload Ca^2+^ influx. These observations suggest that DEX has an antioxidant role against cerebral ischemia-induced oxidative stress in the HIPPO neurons. Sifringer *et al*.^3^ reported that oxidative stress levels in the brains of hyperoxia-exposed developing rats were decreased by DEX treatment. Similarly, Kose *et al*.^19^ found that DEX treatment decreased lipid peroxidation levels in the brains of rats with ischemia-induced brain injuries. The results of this study were supported by the findings of Sifringer *et al*.^3^ and Kose *et al*.^19^.

We found that DEX is a potent blocker of the TRPM2 and TRPV1 currents that are induced by administering CAP through the bath solution and ADPR through the patch pipette. A major source of Ca^2+^ entry during cerebral ischemia is the enhancement of neuronal persistent Ca^2+^ currents through VGCC and NMDA receptors during ischemic conditions^2^. Prolonged opening of Ca^2+^ channels generates a current that contributes to electrophysiological alterations and intracellular Ca^2+^ homeostasis defects^22^, which were similar in the current results. Additionally, the persistent Ca^2+^ current contributes to the propagation of brain injury-induced apoptosis, as indicated by the current results. In recent years, it has become clear that several experimental and pathological conditions can significantly increase the persistence of the TRPM2 and TRPV1 channel currents in the brain^6^,^17^. On this basis, DEX’s inhibition of the TRPM2 and TRPV1 currents contributes to the prevention or limitation of brain injuries during cerebral ischemia. Similarly, Zhao *et al*.^30^ reported that DEX treatment reduced the Ca^2+^ response in astrocytes. In addition, the results of a recent study indicated that DEX treatment decreased histamine-induced Ca^2+^ signaling in cancer cell lines^31^.

Overload [Ca^2+^]_i_ accumulations in cytosol and mitochondria induce apoptosis if the accumulation is not be buffered by the mitochondria^26^,^28^,^29^. Mounting evidence indicates that production of free oxygen radicals and stimulation of apoptotic caspase 3 and 9 pathways are increased in the hippocampus and DRG by increased mitochondrial membrane depolarization (Fig. 8)^5^,^17^. This is because the neurons have poor antioxidant content but high oxygen consumption and polyunsaturated fatty acid content. Hence, antioxidant drugs decrease ROS and apoptosis via regulation of mitochondrial functions in the neuron^5^. Further, DEX has an antioxidant role in neurons^3^,^32^. We have observed clues supporting the hypothesis that cerebral ischemia-induced Ca^2+^ uptake into mitochondria evoked by rises in the concentration of [Ca^2+^]_i_ induces mitochondrial membrane depolarization, excessive free oxygen radical production, and apoptosis values. However, the values were reduced to control levels by TRPM2 and TRPV1 channel blockers. Similarly, it was reported that DEX treatment decreased the number of degenerating cells in brain regions of developing rats exposed to hyperoxia^3^. A recent study suggests the use of DEX to attenuate anesthetic agent-induced apoptosis in neurons^32^. In an ischemia-reperfusion model, *Engelhard et al*.^33^ demonstrated that DEX treatment up-regulated anti-apoptotic proteins and suppressed pro-apoptotic proteins in the brains of cerebral ischemia-induced rats. A similar result was reported in DRG neurons: DEX induced a neuroprotective effect in SCI-related DRG neuron death by inhibiting lipid peroxidation production^18^.

In conclusion, results suggest that DEX treatment reduces cerebral ischemia-induced oxidative stress, cell death, and intracellular Ca^2+^ signaling through inhibition of TRPM2 and TRPV1 in the rat hippocampus and DRG. These findings hold importance and may explain cerebral ischemia-induced HIPPO and DRG injuries, and DEX treatment may indicate for neuroprotective role against apoptotic neuronal death, excessive oxidative stress production, and overload Ca^2+^ influx. The inhibitory effect of DEX in cerebral ischemia-induced TRPM2 and TRPV1 activation should be considered a potential pharmacological target for itching caused by cerebral ischemia-mediated activation of pain and brain injuries.

## Methods

### Animals

Seventy-eight male, 3-month-old, Wistar rats were used in the study. The rats were individually housed under controlled environmental conditions (22 ± 2 °C; 70% humidity), maintained under a 12/12-hour light/dark photoperiod (darkness from 19:00 to 07:00 hours), and fed ad libitum (food and water). The study was performed in Neuroscience Research Center (NOROBAM) of Suleyman Demirel University (SDU) and the study was approved by the Local Experimental Animal Ethical Committee of SDU (protocol number: 09.07.2015-04) in accordance with the National Institutes of Health Guide for the Care and Use of Laboratory Animals and the European Community’s Council Directives (86/609/EEC). All experiments were carried out in accordance with the approved guidelines.

### Study Groups

Two rats were tested pre-study for cerebral ischemia induction. Due to the risk of death, n numbers of operated rat groups which have received a cocktail of ketamine hydrochloride (50 mg/kg) and xylazine (5 mg/kg) administered intraperitoneally (i.p.) were used as n = 12, although the control group n number used was n = 8. The remaining 56 rats are used in 5 groups as follows:

1. First group (n = 8) used as control, and it received i.p. placebo (0.9% w/v physiological saline) at 3, 24, and 48 hours.
2. Second group (n = 12) used as sham control group. The rats were subjected to chirurgical exposure and anesthesia without induction of cerebral ischemia. The rats also received i.p. placebo at 3, 24, and 48 hours.
3. Third group (n = 12) used as DEX group. The anesthesia cocktail was given to the rats in the groups and then DEX (40 μg/ml and DEX hydrochloride, Meditera, Inc, İstanbul Turkey) was given to rats via i.p. injection after 3, 24, and 48 hours^34^.
4. Fourth group (n = 12) used as cerebral ischemia (ISC) group, and ISC was induced through encircling of the rats’ middle cerebral artery. The rats also received placebo via i.p. injection after 3, 24, and 48 hours.
5. Fifth group (n = 12) used as the ISC+DEX group. After ISC induction under the cocktail anesthesia, DEX (40 μg/ml) was given to the rats of the fifth group after 3, 24, and 48 hours^34^.

At the end of 48 hours, a cervical dislocation method was used to scarify the rats. The HIPPO and DRG neurons were obtained as used in previous studies^5^,^6^,^17^.

A part of total DRG and HIPPO neurons were used in patch clamp analyses within 4 hours. The remaining HIPPO and DRG neurons were counted in the CASY Model TTC Cell Counter and Analyzer (Roche Diagnostics Corporation, Indiana, USA), and they were split to sterile cell culture flasks (Greiner Bio-one, Istanbul, Turkey) at a density of 1 × 10^6^ neurons/ml. Some parts of the remaining HIPPO and DRG neurons in 2 ml eppendorf tubes were stored at −85 °C for western blot analyses.

### Induction of Cerebral Ischemia

For anesthesia of the rats, an anesthetic cocktail of ketamine hydrochloride (50 mg/kg) and xylazine (5 mg/kg) was used. The body temperature of the rats was maintained at 37 °C through the use of a heating pad. The right middle cerebral artery was exposed through a ventral midline incision in the neck, carefully isolated from the vago-sympathetic trunks, and loosely encircled with sutures for further occlusion. Following a midline incision, the skull was craniectomized to expose the right common carotid artery. A 3-0 suture was positioned so that it encircled the middle cerebral artery for further occlusion. Cerebral ischemic surgery was performed through occlusion of the right middle cerebral artery and the right middle cerebral artery for 30 min. The sham operated rat was subjected to the same surgical operation but without the occlusion of the artery.

### Electrophysiology

We used whole-cell mode of patch-clamp techniques in EPC10 patch-clamp set (HEKA, Lamprecht, Germany)^17^. Resistances of whole cell recording electrodes were 3–7 MΩ. The standard extracellular bath solutions and pipette solutions were described in previous studies^5^,^17^. The borosilicate patch pipettes were freshly prepared by puller (P-97 Puller, Sutter Instruments Inc., Novato, CA, USA). The intracellular Ca^2+^ concentration was held as 1 μM instead of 0.1 μM in TRPM2 experiments because the channels are activated by presence of high intracellular Ca^2+^ concentration. Holding potential of the patch-clamp analyses in the DRG neurons was -60 mV. Voltage clamp technique was used in the analyses and current-voltage (I-V) relationships were obtained from voltage ramps from −90 to +60 mV applied over 200 milliseconds. All experiments were performed at room temperature (21 ± 1 °C).

In the experiments, TRPM2 channels are gated by ADPR (1 mM in patch pipette) although they were inhibited by N-(p-amylcinnamoyl) anthranilic acid (ACA and 0.025 mM). TRPV1 channels were activated by adding extracellular (in patch chamber) CAP (0.010 mM), and the channels were inhibited administration of capsazepine (CPZ and 0.1 mM) into patch chamber through extracellular buffer. For the analysis, the maximal current amplitudes (pA) in a DRG neuron were divided by the cell capacitance (pF), a measure of the cell surface. The results in the patch clamp experiments are the current density (pA/pF)^35^.

### Intracellular free calcium ([Ca^2+^]_i_) determination

The [Ca^2+^]_i_ concentration was measured UV light-excitable Fura-2 acetoxymethyl ester (Fura-2-AM) as an intracellular calcium ion indicator. The HIPPO and DRG neurons were loaded with 5 μM fura-2 by incubation with (Fura 2-AM) for 45 min at room temperature according to a procedure described elsewhere^25^,^35^. Fluorescence of the [Ca^2+^]_i_ concentration was recorded in a spectrofluorometer (Carry Eclipsys, Varian Inc, Sydney, Australia) from magnetically stirred cellular suspension at 37 °C. In the records, excitation wavelengths were used as 340 and 380 nm although emission wavelength used as at 505 nm. Accumulation of [Ca^2+^]_i_ concentration were calculated from by using the Fura 2-AM fluorescence ratio (340/380 nm). For calibration of the records as nanomolar (nM), the method of Grynkiewicz *et al*.^36^ was used.

### Intracellular ROS production measurement

DHR 123 as cell membrane permanent green florescent dye can easily pass the neuronal cell membranes. It was sequestered by mitochondria. The DHR123 is oxidized Rh123 by intracellular ROS and ratio of Rh123 indicates intracellular ROS production level in the neurons. The DRG and HIPPO neurons were incubated with 20 μm DHR 123 as florescent oxidant dye at 37 °C for 25 min^37^. The Rh123 fluorescence intensities were determined by using an automatic microplate reader (Infinite pro200; Tecan Inc, Groedig, Austria). Excitation and emission wavelengths of the analyses were 488 nm and 543 nm, respectively. The results were expressed as fold-increase.

### Mitochondrial membrane potential (JC-1) analyses

The mitochondrial membrane potential (5,5′,6,6′-tetrachloro-1,1′,3,3′-tetraethylbenzimidazolocarbocyanine iodide, JC-1) was determined by JC-1 dye as described in previous studies^17^,^38^. In health cells, the dye concentrates in the mitochondrial matrix, where it forms red fluorescent aggregates (J-aggregates) and the dye is dispersed throughout the entire cell leading to a shift from red (J-aggregates) to green fluorescence (JC-1 monomers). The JC-1- loaded HIPPO and DRG neurons at 37 °C for 45 min were excited at 488 nm and emission was detected at 590 nm (JC-1 aggregates) and 525 nm (JC-1 monomers). Values were calculated from emission ratios (590/525). The data are presented as fold-increase.

### Assay for apoptosis, caspase 3 and caspase 9 activities

The apoptosis levels were determined in a spectrophotometer (UV-1800 Shimadzu, Kyoto, Japan) by using a commercial kit of Biocolor Ltd. (Northern Ireland). We used a procedure which was described in a previous study^17^. The method is based on loss of asymmetry in membranes of apoptotic neurons.

The determinations of caspase 3 (N-acetyl-Asp-Glu-Val-Asp-7-amino-4-methylcoumarin) and caspase 9 activities in the HIPPO and DRG neurons were determined in the microplate reader (Infinite pro200) by using caspase 3 and caspase 9 (His-Asp-7-amino-4-methylcoumarin). Details of the assays were given in previous studies^37^,^38^. The substrate cleavage was measured at 360 nm (excitation) and 460 nm (emission). Values were calculated as fluorescence units/mg protein and caspase 3 and 9 activities were expressed as fold-increase.

### Western Blot analyses

All Western Blot analyses in the HIPPO neurons were performed using standard procedures are used in the Western Blot analyses of HIPPO and DRG neurons^17^. In the analyses, caspase 9 (p35/p10 Polyclonal Antibody), caspase 3 (p17-specific Polyclonal Antibody), beta actin (polyclonal antibody), PARP1 (polyclonal antibody) were purchased from (Proteintech, USA) although secondary antibodies (Rabbit IgG, HRP-linked whole Ab, from donkey) were purchased from GE Healthcare (Amersham, UK). Visualition of the bands were performed in ECL Western HRP Substrate (Millipore Luminate Forte, USA) by Syngene G:Box Gel Imagination System (UK) and they were normalized against β-actin protein. Obtained values were expressed as relative density over the control level.

### Statistical analyses

All data were represented as means ± standard deviation (SD). The data were analyzed by using 17.0 version of SPSS statistical program (Chicago, Illinois, USA). P ≤ 0.05 was considered to indicate a statistically significant difference. Presence of significance in the five groups was once detected by ANOVA and LSD tests. Then p value levels of significances in the data were analyzed by using Mann-Whitney U test.

## Additional Information

**How to cite this article**: Akpınar, H. *et al*. The neuroprotective action of dexmedetomidine on apoptosis, calcium entry and oxidative stress in cerebral ischemia-induced rats: Contribution of TRPM2 and TRPV1 channels. *Sci. Rep*. **6**, 37196; doi: 10.1038/srep37196 (2016).

**Publisher’s note:** Springer Nature remains neutral with regard to jurisdictional claims in published maps and institutional affiliations.

## References

[b1] Cai Y., Xu H., Yan J., Zhang L. & Lu Y. Molecular targets and mechanism of action of dexmedetomidine in treatment of ischemia/reperfusion injury. Mol Med Rep. 9, 1542–1550 (2014). 24627001 10.3892/mmr.2014.2034

[b2] Kumar V. S., Gopalakrishnan A., Nazıroğlu M. & Rajanikant G. K. Calcium ion-the key player in cerebral ischemia. Curr Med Chem. 21, 2065–2075 (2014). 24372212 10.2174/0929867321666131228204246

[b3] Sifringer M. . Neuroprotective effect of dexmedetomidine on hyperoxia-induced toxicity in the neonatal rat brain. Oxid Med Cell Longev. 2015: 530371 (2015). 25653737 10.1155/2015/530371 PMC4310240

[b4] Nazıroğlu M. New molecular mechanisms on the activation of TRPM2 channels by oxidative stress and ADP-ribose. Neurochem Res. 32, 1990–2001 (2007). 17562166 10.1007/s11064-007-9386-x

[b5] Özdemir Ü. S., Nazıroğlu M., Şenol N. & Ghazizadeh V. Hypericum perforatum attenuates spinal cord injury-induced oxidative stress and apoptosis in the dorsal root ganglion of rats: Involvement of TRPM2 and TRPV1 channels. Mol Neurobiol. 53, 3540–3551 (2016). 26099309 10.1007/s12035-015-9292-1

[b6] Yürüker V., Nazıroğlu M. & Şenol N. Reduction in traumatic brain injury-induced oxidative stress, apoptosis, and calcium entry in rat hippocampus by melatonin: Possible involvement of TRPM2 channels. Metab Brain Dis. 30, 223–231 (2015). 25339252 10.1007/s11011-014-9623-3

[b7] Espino J., Pariente J. A. & Rodríguez A. B. Oxidative stress and immunosenescence: therapeutic effects of melatonin. Oxid Med Cell Longev. 2012, 670294 (2012). 23346283 10.1155/2012/670294 PMC3549369

[b8] Shi Y. Apoptosome: the cellular engine for the activation of caspase-9. Structure 10, 285–288 (2002). 12005427 10.1016/s0969-2126(02)00732-3

[b9] Nazıroğlu M. TRPV1 channel: A potential drug target for treating epilepsy. Curr Neuropharmacol 13, 239–247 (2015). 26411767 10.2174/1570159X13666150216222543 PMC4598436

[b10] Caterina M. J. . The capsaicin receptor: a heat-activated ion channel in the pain pathway. Nature 389, 816–824 (1997). 9349813 10.1038/39807

[b11] Shimizu S., Takahashi N. & Mori Y. TRPs as chemosensors (ROS, RNS, RCS, gasotransmitters). Handb Exp Pharmacol. 223, 767–794 (2014). 24961969 10.1007/978-3-319-05161-1_3

[b12] Nazıroğlu M. & Lückhoff A. A calcium influx pathway regulated separately by oxidative stress and ADP-Ribose in TRPM2 channels: single channel events. Neurochem Res. 33, 1256–1262 (2008). 18259858 10.1007/s11064-007-9577-5

[b13] Cristino L. . Immunohistochemical localization of cannabinoid type 1 and vanilloid transient receptor potential vanilloid type 1 receptors in the mouse brain. Neuroscience 139, 1405–1415 (2006). 16603318 10.1016/j.neuroscience.2006.02.074

[b14] Bai J. Z. & Lipski J. Differential expression of TRPM2 and TRPV4 channels and their potential role in oxidative stress-induced cell death in organotypic HIPPO culture. Neurotoxicology 31, 204–214 (2010). 20064552 10.1016/j.neuro.2010.01.001

[b15] Pecze L., Blum W. & Schwaller B. Mechanism of capsaicin receptor TRPV1-mediated toxicity in pain-sensing neurons focusing on the effects of Na^+^/Ca^2 +^ fluxes and the Ca^2 +^-binding protein calretinin. Biochim Biophy Acta (BBA) 1833(7), 1680–1691 (2013). 10.1016/j.bbamcr.2012.08.018 22982061

[b16] Liu Y. . Normobaric hyperoxia extends neuro- and vaso-Protection of N-Acetylcysteine in transient focal ischemia. Mol Neurobiol. May 13. [Epub ahead of print] (2016). 10.1007/s12035-016-9932-0 27177548

[b17] Kahya M. C., Nazıroğlu M. & Çiğ B. Modulation of diabetes-induced oxidative stress, apoptosis, and Ca^2+^ entry through TRPM2 and TRPV1 channels in dorsal root ganglion and hippocampus of diabetic rats by melatonin and selenium. Mol Neurobiol. [Epub ahead of print]. doi: 10.1007/s12035-016-9727-3 (2016). 26957303

[b18] Can M., Gul S., Bektas S., Hanci V. & Acikgoz S. Effects of dexmedetomidine or methylprednisolone on inflammatory responses in spinal cord injury. Acta Anaesthesiol Scand 53, 1068–1072 (2009). 19519725 10.1111/j.1399-6576.2009.02019.x

[b19] Kose E. A. . Effects of intracisternal and intravenous dexmedetomidine on ischemia-induced brain injury in rat: a comparative study. Turk Neurosurg 23, 208–217 (2013). 23546907 10.5137/1019-5149.JTN.6757-12.0

[b20] Niittykoski M., Haapalinna A. & Sirviö J. Diminution of N-methyl-D-aspartate-induced perturbation of neurotransmission by dexmedetomidine in the CA1 field of rat hippocampus *in vitro*. Neurosci Lett. 28, 95–98 (2000). 10.1016/s0304-3940(00)00811-9 10704751

[b21] Takizuka A. . Dexmedetomidine inhibits muscarinic type 3 receptors expressed in Xenopus oocytes and muscarine-induced intracellular Ca2+ elevation in cultured rat dorsal root ganglia cells. Naunyn Schmiedebergs Arch Pharmacol. 375, 293–301 (2007). 17563882 10.1007/s00210-007-0168-4

[b22] Stoetzer C. . Inhibition of the cardiac Na(+) channel α-subunit Nav1.5 by propofol and dexmedetomidine. Naunyn Schmiedebergs Arch Pharmacol. 389, 315–325 (2016). 26667357 10.1007/s00210-015-1195-1

[b23] Lipski J. . Involvement of TRP-like channels in the acute ischemic response of HIPPO CA1 neurons in brain slices. Brain Res. 1077, 187–199 (2006). 16483552 10.1016/j.brainres.2006.01.016

[b24] Kraft R., Grimm C., Frenzel H. & Harteneck C. Inhibition of TRPM2 cation channels by N-(p-amylcinnamoyl)anthranilic acid. Br J Pharmacol. 148, 264–273 (2006). 16604090 10.1038/sj.bjp.0706739 PMC1751561

[b25] Espino J. . Melatonin reduces apoptosis induced by calcium signaling in human leukocytes: Evidence for the involvement of mitochondria and Bax activation. J Membr Biol. 233, 105–118 (2010). 20130848 10.1007/s00232-010-9230-0

[b26] Shi T. S., Winzer-Serhan U., Leslie F. & Hökfelt T. Distribution and regulation of alpha(2)-adrenoceptors in rat dorsal root ganglia. Pain 84, 319–330 (2000). 10666537 10.1016/s0304-3959(99)00224-9

[b27] Li P. . Cytochrome c and dATP-dependent formation of Apaf-1/caspase-9 complex initiates an apoptotic protease cascade. Cell 91, 479–489 (1997). 9390557 10.1016/s0092-8674(00)80434-1

[b28] Starkov A. A., Chinopoulos C. & Fiskum G. Mitochondrial calcium and oxidative stress as mediators of ischemic brain injury. Cell Calcium 36, 257–264 (2004). 15261481 10.1016/j.ceca.2004.02.012

[b29] Nazıroğlu M. Role of melatonin on calcium signaling and mitochondrial oxidative stress in epilepsy: focus on TRP channels. Tr J Biol 39, 813–821 (2015).

[b30] Zhao Z., Code W. E. & Hertz L. Dexmedetomidine, a potent and highly specific alpha 2 agonist, evokes cytosolic calcium surge in astrocytes but not in neurons. Neuropharmacology 31, 1077–1079 (1992). 1359438 10.1016/0028-3908(92)90111-2

[b31] Yang D. & Hong J. H. Dexmedetomidine modulates histamine-induced Ca(2+) signaling and pro-inflammatory cytokine expression. Korean J Physiol Pharmacol 19, 413–420 (2015). 26330753 10.4196/kjpp.2015.19.5.413 PMC4553400

[b32] Duan X., Li Y., Zhou C., Huang L. & Dong Z. Dexmedetomidine provides neuroprotection: impact on ketamine-induced neuroapoptosis in the developing rat brain. Acta Anaest Scan 58, 1121–1126 (2014). 10.1111/aas.12356 25041263

[b33] Engelhard K. . Effect of the alpha2-agonist dexmedetomidine on cerebral neurotransmitter concentrations during cerebral ischemia in rats. Anesthesiology 96, 450–457 (2002). 11818781 10.1097/00000542-200202000-00034

[b34] Nakano T. & Okamoto H. Dexmedetomidine-induced cerebral hypoperfusion exacerbates ischemic brain injury in rats. J Anesth 23, 378–384 (2009). 19685118 10.1007/s00540-009-0777-9

[b35] Uğuz A. C. . Selenium modulates oxidative stress-induced cell apoptosis in human myeloid HL-60 cells through regulation of calcium release and caspase-3 and -9 activities. J Membr Biol. 232, 15–23 (2009). 19898892 10.1007/s00232-009-9212-2

[b36] Grynkiewicz C., Poenie M. & Tsien R. Y. A new generation of Ca^2+^ indicators with greatly improved fluorescence properties. J Biol Chem. 260, 3440–3450 (1985). 3838314

[b37] Espino J. . Protective effect of melatonin against human leukocyte apoptosis induced by intracellular calcium overload: relation with its antioxidant actions. J Pineal Res. 51, 195–206 (2011). 21470303 10.1111/j.1600-079X.2011.00876.x

[b38] Bejarano I. . Melatonin induces mitochondrial-mediated apoptosis in human myeloid HL-60 cells. J Pineal Res. 46, 392–400 (2009). 19552762 10.1111/j.1600-079X.2009.00675.x

