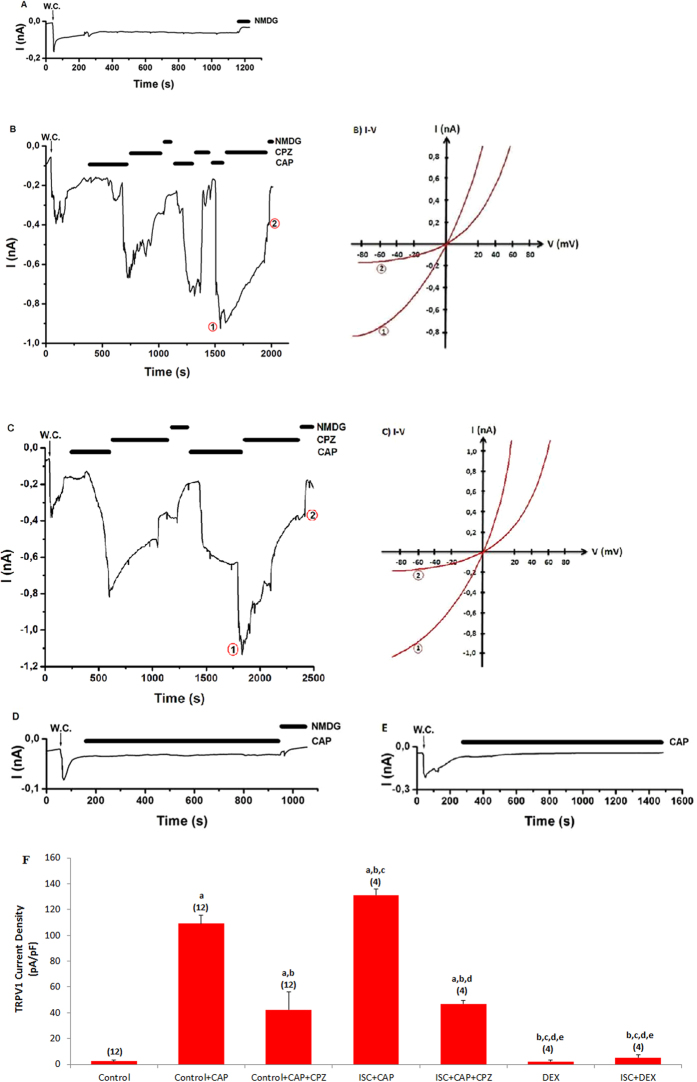# Corrigendum: The neuroprotective action of dexmedetomidine on apoptosis, calcium entry and oxidative stress in cerebral ischemia-induced rats: Contribution of TRPM2 and TRPV1 channels

**DOI:** 10.1038/srep47002

**Published:** 2018-07-04

**Authors:** Hatice Akpınar, Mustafa Nazıroğlu, Ishak Suat Övey, Bilal Çiğ, Orhan Akpınar

Scientific Reports 6; Article number: 3719610.1038/srep37196; published online: 11 22 2016; updated: 07 04 2018

In this Article, the legend of Figure 2 contains errors. The two instances of ‘TRPM2’ should read ‘TRPV1’.

Figure 3 contains errors. Figure 3c was inadvertently duplicated from Figure 4c. Figure 3e is incorrect. The ‘control + ADPR + ACA’ column is missing in Figure 3f. The correct Figure 3 appears below as Figure 1.

Figure 4 contains errors. Figure 4d was inadvertently duplicated from Figure 4e. The correct Figure 4 appears below as Figure 2.

## Figures and Tables

**Figure 1:**
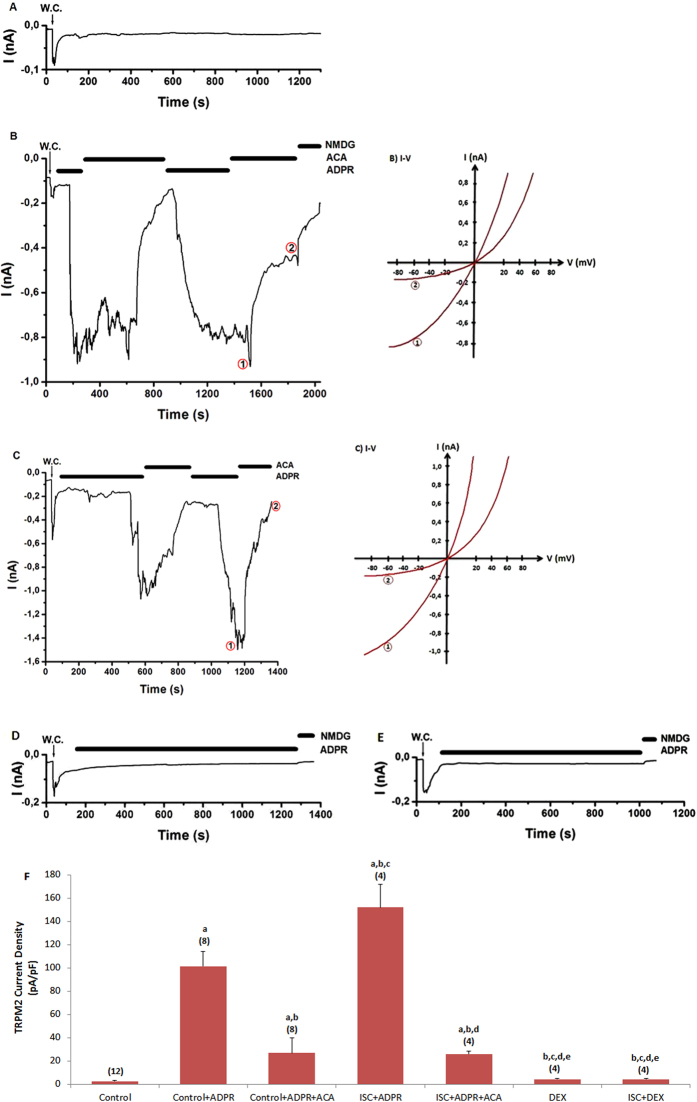


**Figure 2:**